# Community resource referral needs among African American dementia caregivers in an urban community: a qualitative study

**DOI:** 10.1186/s12877-019-1341-6

**Published:** 2019-11-14

**Authors:** Emily M. Abramsohn, Jessica Jerome, Kelsey Paradise, Tia Kostas, Wesley Alexandra Spacht, Stacy Tessler Lindau

**Affiliations:** 1 Department of Obstetrics and Gynecology, The University of Chicago, 5841 S Maryland Ave., MC2050, Chicago, IL 60637 USA; 2 Department of Health Sciences, DePaul University, Chicago, USA; 3 Department of Medicine, Section of Geriatrics & Palliative Medicine, The University of Chicago, Chicago, USA; 4 The University of Chicago Pritzker School of Medicine, Chicago, USA; 5 Departments of Obstetrics and Gynecology and Medicine-Geriatrics, The University of Chicago, Chicago, USA

**Keywords:** Caregiving, Dementia, Qualitative analysis, Race, Self-care

## Abstract

**Background:**

African American caregivers of community-residing persons with dementia are mostly unpaid and have high rates of unmet basic and health needs. The National Alzheimer’s Project Act (NAPA) mandates improved coordination of care for persons with dementia and calls for special attention to racial populations at higher risk for Alzheimer’s Disease or related dementias (ADRD) to decrease health disparities. The purpose of this study is to describe the perceptions of African American caregivers of people with dementia about community resources needed to support caregiving as well as their own self-care.

**Methods:**

Using a qualitative study design, in-depth, semi-structured qualitative interviews were conducted with caregivers (*N* = 13) at an urban geriatric clinic to elicit community resource needs, barriers to and facilitators of resource use and how to optimize clinical referrals to community resources. Caregivers were shown a community resource referral list (“HealtheRx”) developed for people with dementia and were queried to elicit relevance, gaps and insights to inform delivery of this information in the healthcare setting. Data were iteratively coded and analyzed using directed content analysis. Results represent key themes.

**Results:**

Most caregivers were women (*n* = 10, 77%) and offspring (*n* = 8, 62%) of the person with dementia. Community resource needs of these caregivers included social, entertainment, personal self-care and hospice services. Main barriers to resource use were the inability to leave the person with dementia unsupervised and the care recipient’s disinterest in participating in their own self-care. Facilitators of resource use included shared caregiving responsibility and learning about resources from trusted sources. To optimize clinical referrals to resources, caregivers wanted specific eligibility criteria and an indicator of dementia care capability.

**Conclusions:**

African American caregivers in this study identified ways in which community resource referrals by clinicians can be improved to meet their caregiving and self-care needs.

## Background

African American people with Alzheimer’s Disease or related dementias (ADRD) are more likely than others to live in high poverty communities [1] with fewer ADRD-specific support resources [2] and have higher rates of unmet needs than their white counterparts [3]. They are also more likely than others to receive “intensive informal care,” defined as more than 200 h of monthly care from a family or other unpaid caregiver [4]. The 2011 National Alzheimer’s Project Act mandates the improved coordination of care for persons with ADRD and calls for special attention to racial populations at higher risk for ADRD in order to decrease health disparities [5]. Recommended implementation strategies [6] and success measures [7] include ensuring access to ADRD-specific community resources to support caregivers’ ability to provide care while maintaining their own health and well-being. Effective advancement of the NAPA mandates requires engagement with and input from a diversity of ADRD caregivers. To date, there is little evidence of input from African American caregivers on the development of community resource implementation strategies for interventions designed to support caregivers.

**Fig. 1:**
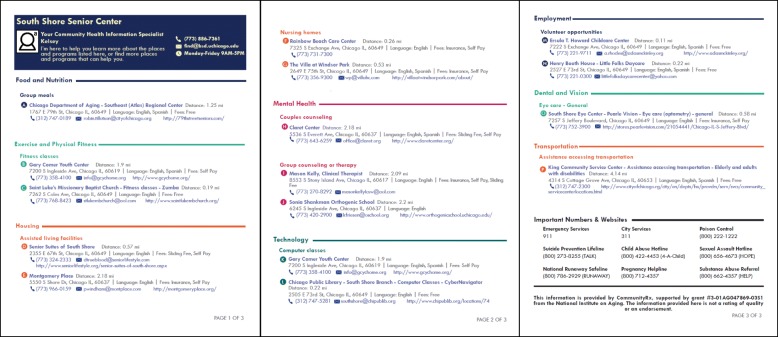
Example HealtheRx for Alzheimer’s Disease and Related Dementias. ^a^The version of the HealtheRx presented to caregivers in this study included a picture of the person in the role of the Community Health Information Specialist

Since 2008, researchers, community leaders and residents, including older adults, have been collaborating on the South Side of Chicago, one of the nation’s largest African American regions [8], to ensure the visibility of and timely access to community resources for self-care and caregiving [9–11]. CommunityRx is a digital community resource referral system that integrates with electronic medical record systems to generate an automated and personalized community resource guide (called a “HealtheRx”) at the point of care (Fig. 1). Developed with support of a 2012–15 Health Care Innovation Award from the U.S. Center for Medicare and Medicaid Innovation (CMMI), CommunityRx has been shown to be an acceptable and scalable intervention to support self-care by systematically connecting people to community resources in a predominantly African American community [9, 12, 13], and is one of few efforts to focus on connecting the ADRD population to community resources. The HealtheRx for people with dementia included resource types indicated by clinical guidelines [14–16], expert opinion and best available evidence [17, 18], including adult daycares, group exercise classes, counseling, volunteer opportunities and transportation services. Although community members were engaged throughout all phases of the original CommunityRx study [10, 19], ADRD caregivers were not specifically solicited for their input and feedback.

While evidence to support the self-care resource needs of dementia caregivers is well established in the literature, the predominance of evidence derives from samples of mostly white caregivers or do not stratify findings by race [20]. Two 2016 studies focused specifically on African American caregivers in the Southern United States and found a general lack of information about or limited access to community resources for caregiving [21, 22]. Building on these findings, this qualitative study on Chicago’s South Side, one of the nation’s largest African American urban communities [8], elicited the perceptions of African American caregivers of people with dementia about community resources needed to support caregiving as well as their own self-care. We further assessed the specific barriers to and facilitators of using community-based resources and caregivers’ insights on how to optimize community resource referrals to better serve their needs.

## Methods

### Research participants

This volunteer sample of research participants included 13 adults who self-identified as African American or Black who were currently providing informal or unpaid care of a person with a diagnosis of dementia. Caregivers were recruited for participation in this study from the University of Chicago Outpatient Senior Health Center located in the South Shore neighborhood (94% African American population) [23]. This site was one of 33 clinical sites that participated in the original CMMI-funded CommunityRx study [9].

### Study design and recruitment

Using a qualitative study design, in depth, semi-structured interviews were conducted with caregivers of community-residing persons with dementia. Caregivers were recruited for this study in-person, either directly from the waiting room or via an on-site, social worker-led support group for caregivers of people with dementia. Eligible participants were 18 years of age or older, English-speaking (as are the vast majority of patients receiving care at this site) and self-identified as the primary caregiver to a person with dementia residing in one of 16 CommunityRx target ZIP codes (a region that includes the primary service area of the Outpatient Senior Health Center). Once introduced to the study, 20 caregivers expressed interest and 16 were eligible for participation. Of these 16 caregivers, 15 were interviewed. The University of Chicago Institutional Review Board approved this study and all participants provided written documentation of informed consent, which included a discussion of the purpose of the research and the risks and benefits of participating.

### Data collection and analysis

In-person, semi-structured qualitative interviews were conducted by two women from the research team (JJ and KP). The interviews elicited information about the following domains: (1) community resource needs for people with dementia and for caregiver self-care, (2) the need for information about hospice resources, and (3) barriers to and facilitators of accessing community resources. Participants were then asked to view a HealtheRx (Fig. 1) for people with dementia, adapted from the CommunityRx project and tailored to include neighborhood resources in which the health clinic was located. Caregivers were asked to describe strategies that would help optimize the content and delivery of community resource guides. The interviews took place in a private room at the Outpatient Senior Health Center and were audio recorded. Data collection concluded when theme saturation was reached across each of the main research domains. Interviews lasted approximately 1 h; participants were provided $30 cash compensation upon completion of the interview.

Data were iteratively coded and analyzed using conventional content analysis [24]. Analysis of the interviews began with a full read of each transcript (EA, STL, JJ and KP) in order to identify recurrent ideas and key themes. A series of meetings were held during which the authors created an initial codebook based on identification of key themes and emerging categories. Coding of interview data began with a second read of each transcript, using the codebook. Any textual data that were identified in the first-pass read of the transcript but not coded using a predefined code were assigned a new code. Throughout the process, two primary coders (JJ, KP) met regularly to discuss and iterate codes and resolve interpretive differences. In order to establish inter-rater reliability, a third researcher (EA) coded the data separately using the final codebook. Two researchers (EA, STL) served as adjudicators when consensus could not be reached by the two primary coders. ATLAS.ti version 7.5.18 (Scientific Software Development, Berlin, Germany) was used for data coding, analysis and textual extraction. Direct quotes are noted with double quotation marks; quotes within a quote are noted with single quotation marks. Quotes are noted by a unique respondent ID and respondent’s age (randomly adjusted by an integer between − 2 and 2 to mitigate the risk of deductive disclosure).

## Results

### Caregiver characteristics

Table 1 describes the sociodemographic, health and caregiving characteristics of caregivers in this study. Caregivers (*N* = 13) ranged in age (44–83 years, median 58 years). Most caregivers were women (*n* = 10, 77%) and many had private insurance for themselves (*n* = 6, 46%). All caregivers were relatives of the person with dementia, but primarily were adult children or grandchildren (*n* = 8, 62%) and spouses (*n* = 4, 31%). Reported duration of caregiving and daily time spent caregiving varied widely (2–25 years, median 5 years, and 1–24 h, median 6.5 h, respectively). Five caregivers (38%) were also providing care for one or more other family members in addition to the person with dementia, including children or grandchildren (*n* = 4, 31% of all caregivers), parents (*n* = 2, 15% of all caregivers) and spouses (*n* = 2, 15% of all caregivers). Most interviews were conducted in the absence of the person with dementia (*n* = 12, 92%).

**Table 1:** Caregiver sociodemographic, health and caregiving characteristics (*N* = 13)

Domain and measure	N (%)
Sociodemographic characteristics	
Age in years^a^ (median, range)	58 (44–83)
Gender	
Women	10 (77)
Men	3 (23)
Insurance^b^	
Private insurance	6 (46)
Private + Medicare	3 (23)
Medicare + Medicaid	2 (15)
Medicare only	1 (8)
Health characteristics	
Self-reported health	
Excellent, very good or good	11 (85)
Fair or poor	2 (15)
Common medical conditions^c,d^	
High blood pressure or hypertension	6 (46)
Osteo- or rheumatoid arthritis	5 (38)
Myocardial infarction, CHF or other heart condition	5 (38)
COPD or asthma	3 (23)
Caregiving characteristics	
Relation to person with dementia	
Adult child or grandchild	8 (62)
Spouse	4 (31)
Sibling	1 (8)
Caregiver lives with person with dementia	8 (62)
Years caring for person with dementia in community (median, range)	5 (2–25)
Hours per day providing care (median, range)	6.5 (1–24)
Provides care for others	5 (38)
Common caregiving tasks^c,e^	
Meal preparation	9 (75)
Medication management/adherence	7 (58)
Accompany to doctor’s visits	6 (50)
Grocery shopping	4 (33)

^a^Caregivers’ ages are not adjusted for anonymity here but are presented in the aggregate

^b^One caregiver refused to answer this question

^c^Responses are not mutually exclusive

^d^Four caregivers reported no comorbidities

^e^One caregiver did not respond to this question

### Need for information about community resources

Caregivers unanimously expressed a strong desire for information about community resource needs for the person for whom they care and for caregiver self-care. With respect to the resources needed to care for the care recipient, caregivers frequently expressed the need for opportunities to socialize. For example, caregivers made comments such as “[We need] some kind of a…place we can go to either meet other people to make connections or to being able to talk to someone who can help us with our challenges.” [ID08, 58]. Caregivers felt that it was particularly important for people with dementia to have the opportunity to socialize with someone other than their caregivers. As one caregiver [ID09, 47] explained, “She probably also needs outside socialization outside of us!” Another caregiver [ID10, 46] stated, “It would be great to have someone … [who] could bring in pet therapy or someone to play music or read to her. You know, have someone to come in and just sit and talk about the weather or the Bulls.” Several caregivers noted that the mood of their loved one with dementia improved in contexts of socialization. For example, one caregiver [ID10, 46] stated, “So just getting her out to do anything that she loves is good. And we take her to plays and the movies. We just try to incorporate and include her in on everything.” Another caregiver [ID08, 58] reported “Socialization. Someone to come, maybe to just come and visit with him. And even that, he fusses if he knows they’re coming, but once they’re there the social graces come in.”

The most common suggestions for community resources that would help fill this need for socialization were support groups, pet therapy, art classes and music therapy. Several caregivers also reported that volunteer opportunities for a person with dementia could provide a meaningful way for them to engage with the world. For example, one caregiver [ID02, 85] shared that her husband “Always talks about volunteering… he would really like to do something and feel like he is being helpful. You know, he thinks about all of the skills that he has or had in the building trade. [He says] ‘I could take a young man and I could show him in the backyard how to do things.’ He would really like to do something, that’s what he talks about all the time. He would like to give back.”

Caregivers identified fitness and dance classes, movie theaters, beauty salons and dining options as important community resources that they could use to address their own self-care needs. Caregivers responded enthusiastically about how these resources could provide a brief respite with comments such as “I love to go to the beauty shop and get my nails done.” [ID03, 59], to “Lord, I’d love to have a massage. You know? Just a day to do, just me. Then go to a movie, dinner, glass of wine. Oh, my God, I’d be in heaven.” [ID12, 50]. And, “Line dancing class! That’s lovely! A lot of seniors, especially African American they do that. They go out line dancing.” [ID08, 58]. A few caregivers described wanting to engage in longer, but still modest, self-care activities that would provide them respite such as a brief vacation or an overnight stay. For example, one caregiver [ID02, 85] stated that it would be useful to know where they could go for “a weekend or overnight. You know I’m not talking about a long cruise or anything! I don’t mean anything like that.” Accessing these resources for self-care, however, often required the caregiver to find outside care for the person with dementia.

All caregivers expressed self-care as necessary to manage the exhaustion of caregiving. As one caregiver [ID08, 58] put it, “Everything we do…is for our spouses [the care recipient]. I know we took that vow… But we need…something for us. Ok?” Another caregiver [ID13, 54] stated, “Work is my therapy. I get more of a rest at work than I do at home.” More than half of the caregivers we interviewed expressed distress over the demands of caregiving, as they discussed their need for self-care. Emblematic of these comments was the caregiver [ID02, 85] who stated, “Right now I am miserable, I can’t—I haven’t been on a vacation since 2008. I just haven’t—any time anybody comes to help, they are relieving my husband [the person for whom she was caring].”

### Awareness of and need for hospice care resources

Caregivers were also asked about their awareness of and need for hospice care resources. Most of the caregivers reported being aware of what hospice was and had a positive impression of the care provided by hospice services. Despite a general awareness of hospice care, many of the caregivers reported being uncertain about the specific kind of services provided by hospice. For example, one caregiver [ID12, 50] asked “…I didn’t know how intensive [hospice] was. Do they come and they bathe them? Do they just come and see that everything is okay? Do they clean while they’re there? Do they do more than … make sure a meal is made for them? Do they do more than just administer medication?” Another caregiver [ID02, 74] asked: “I’m wondering what that’s [hospice] like…I think it’s something that should be discussed, because a lot of people have not been a part of it, so they don’t know.”

Only a few caregivers noted neutral or negative impressions of end-of-life care; for example, one caregiver [ID15, 56] stated: “There was a spiritual counselor that would come and talk to my mom. One day he asked my mom if she was ready to die. And I really hated that. I hated the word hospice.” Not one caregiver in our study reported that it was inappropriate for healthcare providers to discuss end-of-life care options for the person with dementia for whom they were caring.

### Barriers to accessing community resources

Although caregivers were consistent in their desire for information about community resources that would support opportunities for socialization for people with dementia as well as their own self-care, they also reported significant barriers to accessing those resources.

The most commonly reported barrier to accessing community resources, both for socialization for the person with dementia and for caregiver self-care, was the inability to leave the person with dementia unsupervised (Table 2). Typical of these responses was the caregiver [ID05, 67] who stated, “I really get nervous about leaving her at home by herself.” Caregivers often stressed the close attention that their loved ones needed, as with the caregiver [ID03, 59] who explained, “My mother will go in the refrigerator and pick up anything, eat it, it be done or undone, you know. And you just gotta make sure, you know. Cause she’ll pick up some hamburger undone and eat it there—come on now. So, you have to watch her, you have to keep close contact on her.”

**Table 2:** Quotes exemplary of caregivers’ inability to leave person with dementia unsupervised

	Caregiver characteristics
Inability to leave person with dementia unsupervised as a barrier to their socialization	
“She’s gonna have to have love and nothing but lovin’ people around her. Because she would ask you the same question. You gonna get questions from her. As of late, she asks you the same question 15 times. You know, and it takes a lot of patience.”	ID03: 59-year-old daughter
“If I was to leave and be gone for a week, I don’t know what she would do or who she would talk to.”	ID05: 67-year-old spouse
“There is days where, or there is times where during the day I have to take a deep break … and I have to remember that she has Alzheimer’s, she has dementia. I have to remember that. Because some of the things she says you know, it’s a constant. It’s a constant. And then every day is the same.”	
Inability to leave person with dementia unsupervised as a barrier to caregiver self-care	
“My mother will go in the refrigerator and pick up anything, eat it, it be done or undone, you know. And you just gotta make sure, you know. Cause she’ll pick up some hamburger undone and eat it there—come on now. So, you have to watch her, you have to keep close contact on her.”	ID03: 59-year-old daughter
“We’ll have a six pack, and you’ll look around and she will have grabbed the bottles of pop and she’ll drink three of them. By the time you look back again she’s got the other two! I say, ‘I can’t turn my back on you!’ You know? [laughs]. You can’t do it!”	ID04: 64-year-old son
“I’m runnin’ on trial by error. But I’m also now runnin’ on like ‘Nahhhh Imma go with you.’ And if she wants to go somewhere, then we go. So I don’t have a schedule.”	ID05: 67-year-old spouse
“But right now, I really get nervous about leaving her at home by herself. Not so much now, because she hasn’t gotten that urge to go. She wants to go somewhere, but she hasn’t gotten that urge to just get up and go on her own.”	
“In order for me to feel safe to leave him with the kids, they’d all have to be asleep. I’d have to make sure that no one would be able to wake up until I came back upstairs because once I get home I can’t leave the kids and [NAME] by themselves. It worries me that, first that he might do something like turn on the stove, you know? But second, that if, because they are children that are 7 and 3, if they do anything that aggravates him, will he become violent. So I can’t, and I have to.”	ID12: 50-year-old spouse

Several caregivers also expressed more serious concerns about the safety of family members who would be left alone with the person with dementia. For example, one caregiver [ID12, 50] stated, “In order for me to feel safe to leave him with the kids, they’d all have to be asleep. I’d have to make sure that no one would be able to wake up until I came back upstairs because once I get home I can’t leave the kids and [care recipient] by themselves. It worries me that, first that he might do something like turn on the stove, you know? But second, that if, because they are children that are 7 and 3, if they do anything that aggravates him, will he become violent.”

With regard to socialization, caregivers identified a range of barriers that prevented their loved ones with dementia from taking advantage of extant opportunities, including mental confusion, mobility issues and lack of interest. For example, one caregiver [ID05, 67] described trying to take his wife to a swim class and reported, “So we were going to the [center name] up there for like a swimming class but she couldn’t—she couldn’t understand what—she couldn’t follow directions. So I have to be right there with her, for her to do what everybody else is doing. I mean, it’s not like she would even look around and see what everybody else is doing so…” Caregivers also discussed changes in care recipients’ mobility as something that prevented the person with dementia from accessing community resources. For example, one caregiver [ID08, 58] explained, “He used to walk 3 miles a day. But he can’t anymore because he’s having balance issues.”

Lack of interest on behalf of the person with dementia was also seen by caregivers as a significant barrier to accessing community resources. As one caregiver [ID13, 54] explained, “I’m thinking, ‘Oh there’s nothing out near me, everything is in [neighborhood] or up north.’ But that’s not true, there are things close to me but I just haven’t [had the time]…the need [for self-care] might be there but I just keep getting that resistance from my father.”

### Facilitators of accessing community resources

Almost all caregivers noted that being able to at least occasionally rely on other caregivers to take care of or supervise the care recipient helped to facilitate their use of community resources. Arrangements for additional caregiving included formal agreements with other family members or paid caregivers as well as less frequent and unscheduled offers of help. Shared caregiving responsibilities facilitated their use of community resources by allowing them time to support the care recipient’s basic daily needs, including grocery shopping, laundry or to pick up prescriptions or other medications. As one caregiver [ID04, 64] noted, “[It’s] 24 hours a day. I’m there every day… I maybe take a day off when my sisters come in to town. I maybe take 4 hours and go food shopping, we have our own laundry, I take care of that and many other particulars that have to be taken care of for her.” Another caregiver [ID06, 66] reported “No, no [I don’t have time to use community resources] unless my sisters be there with my mom. Or my brother or someone while I go and pick up Ma’s medication.”

Additionally, caregivers reported that sharing caregiving responsibilities facilitated information exchange about community resources, especially among family members. This type of “word of mouth” exchange was repeatedly cited as the most important source of information about the resources necessary to take care of people with dementia and usually came from a trusted source. Most often caregivers reported that information about community resources was shared among caregivers (especially while attending dementia caregiver support groups), and between the caregiver and other family members including siblings, parents, children and extended family. A typical comment describing the way information about dementia passed among family members was: “So now, I do all of that and my sister who doesn’t have time to go to the meetings, because she is working, but I talk to her about it. So I pass on what I’ve learned. Some of the things that we encounter, I’m like, ok I’m more familiar with this. I’ve read about this. We’ve talked about this in group. Ok I kinda know what’s going on. So, that’s helped me a lot.” [ID9, 47]. Another caregiver [ID13, 54], commenting on the importance of trading information about dementia stated: “Usually I talk to people that I believe are in the same situation. I see what works for them. I talk to family. I just—where I used to be really very reserved, now if I have a need I try to speak up and I try to ask questions. You never know if you don’t ask.” Sometimes the caregiver’s source was a member of the person with dementia’s healthcare team. For example, one caregiver [ID10, 46] stated, “You gotta listen. If you have a great quality physician that has a caring heart, she makes great recommendations.”

### Strategies to optimize community resource access and use

Caregivers were asked to view a HealtheRx for people with dementia that was tailored to include community resources in the neighborhood in which the health clinic was located. Caregivers were asked to describe how this sample community resource guide could be improved to support their care for a person with dementia. Two suggestions were common: to include detailed eligibility criteria on the guide and to indicate which service providers are trained in dementia care needs.

In discussing the importance of including health-related eligibility criteria for dementia care services, one caregiver [ID13, 54] expressed her concern in the following way: “‘Cuz [sic] I’ve heard at various places [nursing home] they won’t — they wanna take a person who can still wash themselves and put on their clothes and stuff. And if they are too far gone, they don’t wanna take em. Ok? So, cuz like a person with my father’s personality—I’m gonna have to wait until something dire happens.”

Caregivers also asserted that including information about financial eligibility on community resource guides was important. For example, one caregiver [ID02, 85] expressed concern in the following way, “I never apply for things, like trying to get something for free or something for nothing. Where do you go when you are willing to pay on a sliding scale?” Another caregiver [ID12, 50] shared this sentiment: “Are there programs like the TANF [Temporary Assistance for Needy Families] program where you can apply if you are on a certain temporary family assisted fee? If you qualify for that, how do you qualify for it? Who do you contact for it? And they will give you certain monies to help you out...?”

In addition to eligibility criteria, caregivers consistently stressed that it was important that service providers be trained in dementia care needs. For example, looking at the sample community resource guide, one caregiver [ID05, 67] stated, “I don’t think if anywhere that we went, if they weren’t trained in recognizing the dementia and the habits of the people that have it…if they weren’t trained in it, then that’s not a place where somebody might wanna go.”

One caregiver [ID05, 67] reported experiencing a hostile interaction with untrained service providers at a community-based organization where the person with dementia was misunderstood: “They have all kind of programs over there. But, for a person with dementia, the [early] onset dementia or the Alzheimer’s, I’m finding that some people don’t really know anything about it. It’s not like a well-known thing… And a lot of people that I come across when I’m with her… sometimes she says things and people say ‘No ma’am, no.’ You know they haven’t, they haven’t got a clue!” In contrast, one caregiver [ID09, 47] reported a positive experience with a community-based art class designed for people with dementia and their caregivers. She stated “The instructor is fantastic. She’s clearly got a lot of experience working with a range of people. She is just very personable.”

## Discussion

Urban-dwelling African American caregivers of home-dwelling people with dementia in the current study need more and better quality information about caregiving and self-care resources in their community. This finding corroborates evidence from prior studies that have focused on caregiving-specific needs [3, 25, 26] and adds knowledge about the kinds of resources these caregivers need for their own wellness and self-care. African American caregivers in our study specifically identified a desire for volunteer opportunities that could include the care recipient. The idea to enable volunteerism among home-dwelling people with dementia is unique to the caregiving literature and consistent with Maslow’s Theory of Human Motivation, which specifies the human need for self-esteem and self-actualization [27].

The inability to leave the person with dementia unsupervised is a common concern among caregivers [26, 28, 29], and was the main barrier to community resource use identified in this study. Although prior qualitative studies of African American dementia caregivers living in both rural and urban areas found a lack of knowledge [22] or limited availability [21] of local resources as common barriers to self-care, neither surfaced as a major barrier in this study. Rather, caregivers focused on the concern that local businesses and organizations were not well informed about or prepared to serve people with dementia. This finding resonates with that of prior studies, including one community-based effort undertaken by the University of Kentucky to better understand barriers to accessing dementia services by African American people with dementia and their caregivers. Through qualitative focus groups with key people in the community, they learned of the stigma associated with having memory problems and other mental health issues among African American people, often resulting in hesitancy to access care [30].

Shared caregiving responsibilities, while often informal and described as inadequate in terms of providing relief for most caregivers, surfaced as the main facilitator of community resource use and promoted information exchange. Similarly, Oliveira et al. (2018) identified shared caregiving responsibilities as a facilitator of caregivers’ quality of life by providing respite and facilitating decision making [31]. In the current study, learning about community resources from a trusted person also promoted resource use, suggesting that this information should be delivered to caregivers by a trusted person and that caregivers would value a tool that would enable them to share the information with others. Interestingly, in two prior studies of the CommunityRx intervention, we found that half of people who received the intervention shared the information with others [9, 32].

By asking caregivers for feedback on the HealtheRx for people with dementia, we uncovered demand for a broader variety of community resources than identified in prior studies. Activation of social, entertainment and personal self-care services and activities that could include both the caregiver and the care recipient and provide caregiver respite requires engagement and awareness among proprietors in sectors beyond human and social services. Beauty salons and barber shops, among the most prevalent business types on Chicago’s South Side [11], have been successfully engaged in health promotion efforts to improve hypertension and cancer screening rates, especially in African American communities [33–35], suggesting the possibility that these types of businesses could be engaged to support people with dementia and their caregivers. Education and mobilization of these economic sectors to better support caregivers and people with dementia aligns with the call for more dementia-capable and dementia-friendly communities [36].

Caregivers also endorsed a need to understand which community resources are indicated, or for which services the care recipient is eligible, at different stages of dementia illness. This finding resonates with a qualitative analysis by Granbo and colleagues who identified the need for resources that provided “individualized support” for people with dementia that took into account their current physical abilities as opposed to providing “passive care,” exemplified as resources that were not meaningful or stimulating for the person with dementia [26]. Similarly, Potter and colleagues found African American caregivers were less likely to use community supports early in the course of the disease but were open to learning about resources for future needs [37]. Although prior caregiver intervention studies [3, 38, 39] included dementia education and community resource information components, none describes providing stage-specific resource referral information.

Furthermore, few prior studies have elicited input from caregivers of people with dementia on when and how to deliver information about or referrals to hospice care. Prior evidence suggests that African American caregivers may be less receptive to or more skeptical of hospice care [40, 41]. Our study specifically probed caregivers on the topic of end-of-life care, including hospice, revealing a substantial unmet need for information and no resistance to discussing the topic. The Alzheimer’s Association recommends that conversations with families about end-of-life care begin at diagnosis and continue throughout the progression of the disease, but recognizes that end-of-life care discussions are often not initiated until the patient’s admission to assisted living or other formalized care [42]. This recommendation is particularly pertinent for African Americans, who bear a higher disease burden, may be less likely to access end-of-life care options and are more likely to rely on informal caregivers for support [41, 43]. Including end-of-life resources on the HealtheRx for people with dementia is a potential tool to help facilitate and normalize these conversations earlier in the course of the disease.

### Limitations

The findings of this single site study should be considered in light of certain limitations. Findings may not be generalizable to other populations, including African American caregivers living in higher income or more diverse or rural communities. The study was conducted at a specialized geriatric care clinic; even still, caregivers indicated a broad range of unmet needs. Although the sample size was small, it was diverse in terms of age, caregiving intensity and relationship of the caregiver to the person with dementia, demonstrating similar information and resource needs from various ADRD caregivers’ perspectives. Because some recruitment occurred in a group setting, we are unable to fully quantify the number of caregivers approached for participation and reasons for deciding not to participate in the study. Theme saturation was reached and all participants were currently providing care, which should limit recall bias.

## Conclusion

Informal caregiving presents challenges to ADRD caregivers’ capacity to manage their own health and well-being. This study identifies ways that community resource referrals by clinicians can be improved to meet African American ADRD care recipient and caregiver needs. For African American ADRD caregivers to benefit from community resource referral interventions, this study suggests the information should be delivered by a trusted source, eligibility criteria should be clear and community-based service providers should deliver dementia-capable services.

## Data Availability

The datasets generated and/or analyzed during the current study are not publicly available due to the qualitative nature and to protect the confidentiality of the participants.

## Abbreviations

ADRD: Alzheimer’s disease and related dementias
NAPA: The National Alzheimer’s Project Act
